# *MAPRE2* mutations result in altered human cranial neural crest migration, underlying craniofacial malformations in CSC-KT syndrome

**DOI:** 10.1038/s41598-021-83771-3

**Published:** 2021-03-02

**Authors:** Cedric Thues, Jorge S. Valadas, Liesbeth Deaulmerie, Ann Geens, Amit K. Chouhan, Ramon Duran-Romaña, Joost Schymkowitz, Frederic Rousseau, Michaela Bartusel, Rizwan Rehimi, Alvaro Rada-Iglesias, Patrik Verstreken, Hilde Van Esch

**Affiliations:** 1 Laboratory for the Genetics of Cognition, Department of Human Genetics, Center for Human Genetics, KU Leuven, Herestraat 49, 3000 Leuven, Belgium; 2 VIB Center for Brain & Disease Research, KU Leuven, Herestraat 49, 3000 Leuven, Belgium; 3 Department of Neurosciences, Leuven Brain Institute, KU Leuven, Herestraat 49, 3000 Leuven, Belgium; 4 Switch Laboratory, VIB Center for Brain and Disease Research, Herestraat 49, 3000 Leuven, Belgium; 5 Switch Laboratory, Department of Cellular and Molecular Medicine, KU Leuven, Herestraat 49, 3000 Leuven, Belgium; 6 Center for Molecular Medicine Cologne (CMMC), University of Cologne, Robert-Koch-Strasse 21, 50931 Cologne, Germany; 7 Department of Biology, Massachusetts Institute of Technology, 31 Ames St., Cambridge, MA 02142 USA; 8 Cologne Excellence Cluster for Cellular Stress Responses in Aging-Associated Diseases (CECAD), University of Cologne, Joseph-Stelzmann-Strasse 26, 50931 Cologne, Germany; 9 Institute of Biomedicine and Biotechnology of Cantabria (IBBTEC), CSIC/Universidad de Cantabria, Albert Einstein 22, 39011 Santander, Spain

**Keywords:** Developmental biology, Genetics, Stem cells, Medical research

## Abstract

Circumferential skin creases (CSC-KT) is a rare polymalformative syndrome characterised by intellectual disability associated with skin creases on the limbs, and very characteristic craniofacial malformations. Previously, heterozygous and homozygous mutations in *MAPRE2* were found to be causal for this disease. *MAPRE2* encodes for a member of evolutionary conserved microtubule plus end tracking proteins, the end binding (EB) family. Unlike MAPRE1 and MAPRE3, MAPRE2 is not required for the persistent growth and stabilization of microtubules, but plays a role in other cellular processes such as mitotic progression and regulation of cell adhesion. The mutations identified in *MAPRE2* all reside within the calponin homology domain, responsible to track and interact with the plus-end tip of growing microtubules, and previous data showed that altered dosage of MAPRE2 resulted in abnormal branchial arch patterning in zebrafish. In this study, we developed patient derived induced pluripotent stem cell lines for MAPRE2, together with isogenic controls, using CRISPR/Cas9 technology, and differentiated them towards neural crest cells with cranial identity. We show that changes in MAPRE2 lead to alterations in neural crest migration in vitro but also in vivo, following xenotransplantation of neural crest progenitors into developing chicken embryos. In addition, we provide evidence that changes in focal adhesion might underlie the altered cell motility of the MAPRE2 mutant cranial neural crest cells. Our data provide evidence that MAPRE2 is involved in cellular migration of cranial neural crest and offers critical insights into the mechanism underlying the craniofacial dysmorphisms and cleft palate present in CSC-KT patients. This adds the CSC-KT disorder to the growing list of neurocristopathies.

## Introduction

In 2011, Wouters et al., described two unrelated patients with identical clinical manifestations, consisting of intellectual disability (ID) associated with characteristic dysmorphic features, including symmetrical skin creases on the limbs, cleft palate, facial dysmorphism, short stature and borderline microcephaly. The term “circumferential skin creases, Kunze type” (CSC-KT, MIM 616734) was coined and through whole-exome sequencing pathogenic mutations in *TUBB* (MIM 191130) and in *MAPRE2* (MIM 605789), were identified as the underlying genetic cause^1,2^. Both pathogenic homozygous missense mutations as well as heterozygous nonsense mutations in *MAPRE2* (microtubule associated protein member 2) were found as causal for the disease^1^.

The dynamic behavior of microtubules (MT) is regulated by several factors, including the binding of associated proteins, and by transient interactions of the GTP cap with members of a family of proteins termed + TIPs. Among this diverse group of evolutionarily conserved microtubule plus-end tracking proteins, the best characterized is the end-binding (EB) family of proteins, to which *MAPRE2* belongs^3^. Proteins of this family bind to the microtubule plus-ends and recruit a network of other + TIPs that regulate the interactions of MT with multiple cellular structures and organelles^4,5^. Unlike MAPRE1 and MAPRE3, MAPRE2 is not required for the persistent growth and stabilization of MT^6,7^. However, MAPRE2 plays a role in the reorganization of MT during early apical-basal differentiation in epithelia, regulation of cell adhesion, mitotic progression and genome stability^8–12^.

The *MAPRE2* mutations that we identified affect the highly conserved calponin-homology (CH) domain, which is responsible to track and interact with the plus-end tip of growing MT^1^. Previous in vitro microtubule-polymerization assays showed that the missense mutations lead to a strongly increased affinity and dwell time at the MT, indicating a gain-of-function effect^1^. Experiments performed in zebrafish embryos showed that knock-down of *MAPRE2* resulted in craniofacial defects, consisting of aberrant formation of the angle between early bilateral cartilaginous structures and a significant delay in rostro-caudal ceratobranchial arch patterning. Furthermore, in vivo rescue experiments lead us to speculate that *MAPRE2* mutations exhibit a “Goldilocks effect” whereby, at least for the maturation of the branchial arches, either overactive or insufficient protein ultimately leads to the same clinical pathology^1^.

Neural crest cells are a transient, embryonic population of cells that delaminate from the roof of the neural tube during neurulation. Subsequently, these cells undertake the longest migration of any embryonic cell type, migrating throughout the embryo to form a wide array of different cell lineages, contributing to nearly every organ in the body^13^. Proper migration of cranial neural crest is essential for normal facial and palatal development, as this special population of cells constitutes the majority of skeletal and connective tissues in the face^14^. Given that CSC-KT patients display cleft palate associated with highly specific facial dysmorphism, we hypothesize that MAPRE2 plays a crucial role in cranial neural crest migration during facial development. Therefore, we generated a human induced pluripotent stem cell (iPSC) model and derived cranial neural crest cells (CNCC). We find that changes in MAPRE2 alter neural crest migration in vitro, potentially due to alterations in focal adhesion. To determine if these migration defects also occur in vivo, we performed xenotransplantation of human derived *MAPRE2* haploinsufficient cranial neural crest progenitors into developing chicken embryos. This shows impaired migration into the craniofacial structures, a feature that could explain the facial anomalies seen in patients. Our work suggests that neural crest migration defects are a key aspect of CSC-KT.

## Results

### Generation of MAPRE2 N68S isogenic control and patient mutation Q152* knock-in lines

**Figure 1:**
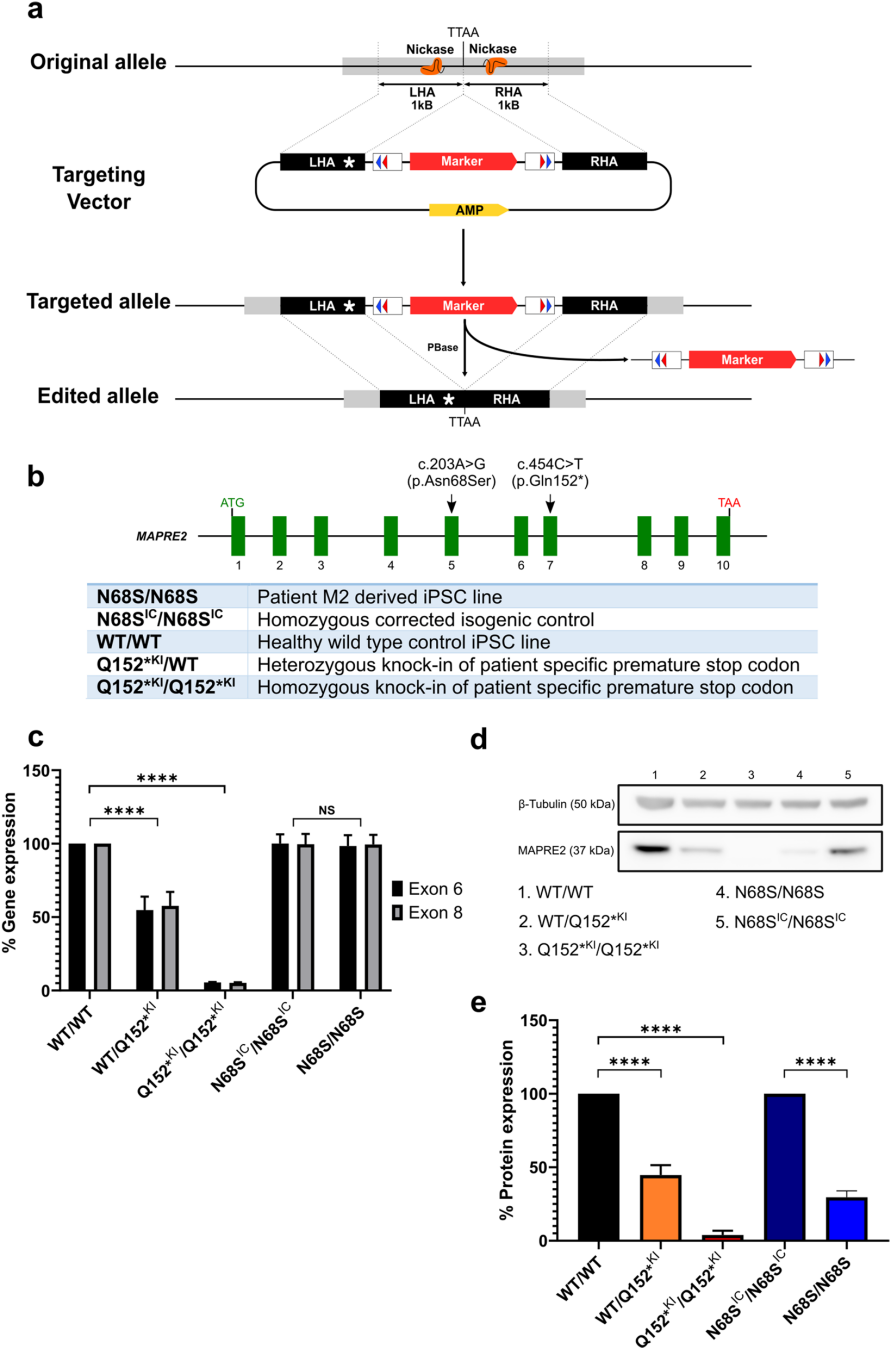
Overview of CRISPR-Cas9 gene editing strategy and MAPRE2 expression in newly generated cell lines. (**a**) General overview of CRISPR/Cas9 gene editing strategy adapted from Yusa et al.^17^. (**b**) Overview of the different MAPRE2 genotypes generated. (**c**) RT-qPCR mRNA expression levels of MAPRE2 exon 6 and exon 8 normalized to GAPDH in non-differentiated iPSCs (n = 3). (**d**) Immunoblotting from non-differentiated iPSC whole cell protein lysate against MAPRE2 and β-Tubulin. Full length blot is included in Supplementary Fig. S3, f. (**e**) Western blot MAPRE2 protein expression levels normalized to β-Tubulin in non-differentiated iPSCs (n = 5). Q152*^KI^ mutants are normalized to WT/WT and N68S/N68S is normalized to N68S^IC^/N68S^IC^.

We previously showed that the homozygous missense mutation p.Asn68Ser (N68S) results in increased protein activity, while the heterozygous nonsense mutation p.Gln152* (Q152*) results in haploinsufficiency of the protein^1^. We chose both mutations for further modelling. We generated a patient derived induced pluripotent stem cell line of the CSC-KT patient (M2), who carries a homozygous p.Asn68Ser (N68S) missense mutation^1^. This iPSC line, named N68S/N68S, was fully validated (Supplementary Fig. S1). Subsequently, we generated an isogenic control line, N68S^IC^/N68S^IC^, correcting both mutant alleles using CRISPR/Cas9 technology (Fig. 1a, Supplementary Fig. S2). Since we were unable to acquire biological material from the patient (M1) who carries the heterozygous nonsense mutation p.Gln152* (Q152*), we introduced this premature stop codon (located in exon 7 of *MAPRE2*) into a wild type in-house human IPSC line (BJ1^15^), again by applying CRISPR/Cas9 technology (Supplementary Fig. S2). This resulted in the heterozygous knock-in iPSC line, Q152*^KI^/WT, similar to the genotype of patient M1. We also obtained an iPSC line where both *MAPRE2* alleles were targeted, Q152*^KI^/Q152*^KI^ representing a complete knock-out for the protein.

In order to reduce CRISPR/Cas9 induced off-target cuts, we used the D10A Cas9 mutant nickase with a pair of offset sgRNA’s on the complementary opposite strand of the target site. Individual nicks are efficiently repaired by the high fidelity base excision repair pathway, while nicking of opposite strands within 100 base pairs will lead to a site specific double strand break^16^. In addition, to obtain clean and “scar less” edits we used this double nicking CRISPR-Cas9 system in combination with the PiggyBac transposon system (Fig. 1a and Supplementary Figs. S2, S3 and S4)^17^. A summary of all available *MAPRE2* genotypes is shown in Fig. 1b.

Next, we evaluated the expression of *MAPRE2* in our different iPSC lines by RT-qPCR and Western blotting. As hypothesized, a reduction in *MAPRE2* mRNA levels of 95% and 45% was observed in the Q152*^KI^/Q152*^KI^ and WT/Q152*^KI^ mutants, respectively (One-way ANOVA, Tukey test, P < 0.0001 and P < 0.0001), using primer sets in exon 6 and in exon 8 of the gene (Fig. 1c). Western blotting analysis also showed a reduction of 96% and 55% in protein expression levels, respectively (One-way ANOVA, Tukey test, P < 0.0001 and P < 0.0001) (Fig. 1d,e and Supplementary Fig. S3). No reduction in mRNA levels was observed for the N68S/N68S mutant compared to its isogenic control line, N68S^IC^/N68S^IC^ (Unpaired t test, P = 0.9772). However, we unexpectedly observed a great reduction in protein expression (70%, Unpaired t test, P < 0.0001) for the N68S/N68S mutant compared to the isogenic control (Fig. 1c–e), which can originate from a reduced protein stability.

### Residue 68 in MAPRE2 is highly conserved and the p.Asn68Ser mutation causes protein instability

**Figure 2:**
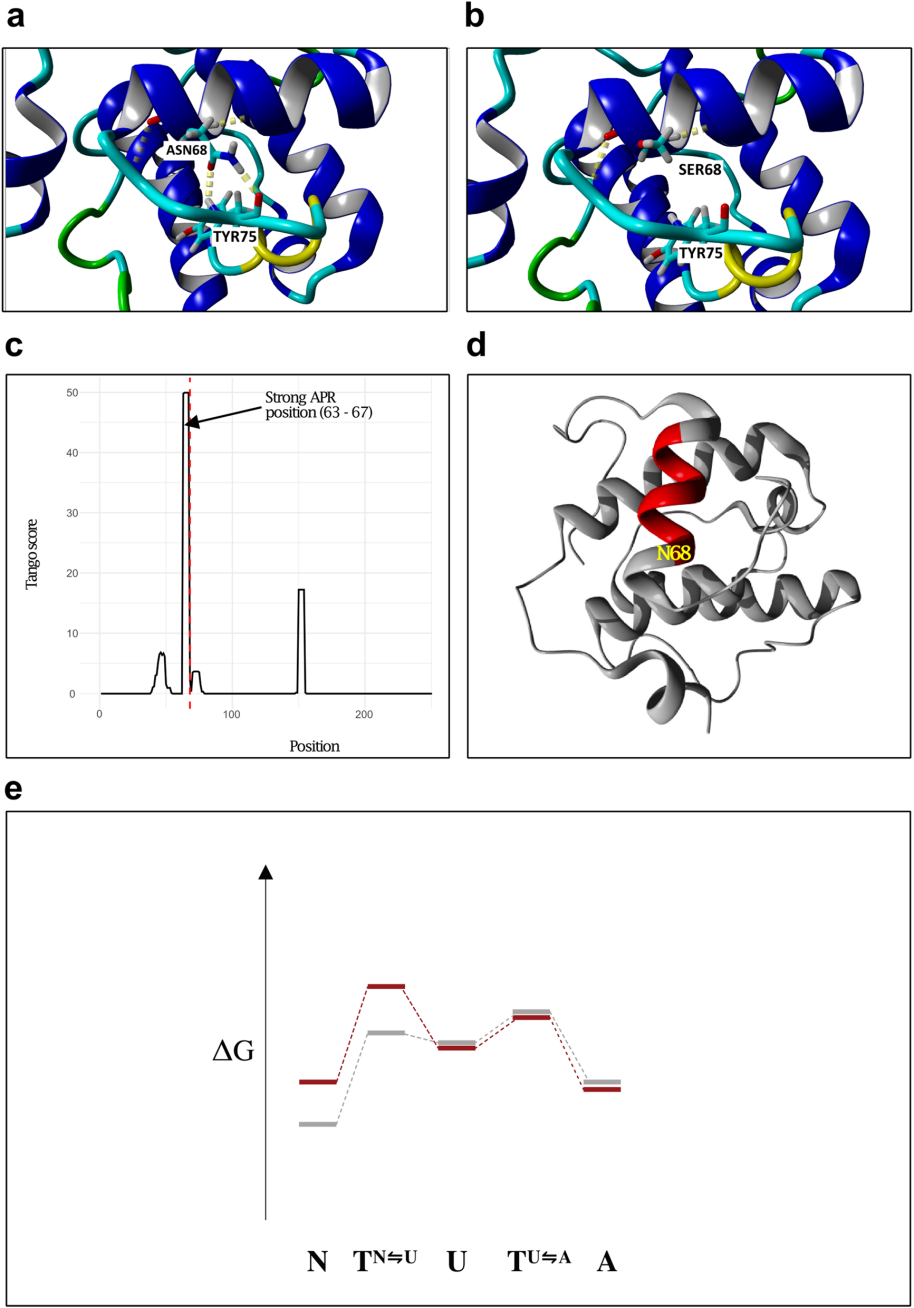
Modelling of the MAPRE2 N68S mutation. (**a**, **b**) The size and hydrophobicity difference between (**a**) wild type (Asn) and (**b**) mutant (Ser) residue makes the mutant unable to form the same hydrogen bonds. (**c**) Aggregation propensity prediction TANGO scores per residue for wild type MAPRE2. Vertical red line indicates the position of N68. (**d**) Model structure of MAPRE2 with the strong APR colored in red. (**e**) Energetic landscape of the mechanism proposed. N68S mutation is indicated in red and wild type energy landscape in grey. N68S thermodynamically destabilizes and kinetically slows down the folding reaction, resulting in higher energies for N and T^N⇋U^ and less natively folded protein (*N* native fold, *U* unfolded state, *A* aggregated state, *T* transition state).

To further explain this significant reduction in protein expression of MAPRE2 in the N68S/N68S line, we first looked at the conservation of residue 68 in a set of 25 reviewed proteins of the MAPRE family by building a multiple sequence alignment with Clustal Omega^18^. This set includes proteins from organisms with high evolutionary distances from humans, such as *Arabidopsis thaliana* or *Saccharomyces cerevisiae*. Residue position 68 has a 100% conservation in this protein family, suggesting that the residue is structurally and/or functionally very important (Supplementary Fig. S5). The exact 3D-structure of the MAPRE2 protein is unknown. Hence, we built a model based on a homologous structure using the Swiss-model tool^19^. The best template based on quality, coverage and sequence identity was 1VKA from *MAPRE1*. This model has a sequence identity of 75.18% and covers residues 45–174 (the whole calponin homology domain) (Supplementary Fig. S5). The thermodynamic stability of the mutant was computed using the atomic forcefield FoldX^20^. Based on FoldX calculations, the difference in free energy of the mutation (ΔΔG) was 1.35 kcal/mol. This implies that the N68S mutation reduces protein stability. More specifically, this reduction in stability can be explained by the loss of two hydrogen bonds of the mutant residue with Tyr 75 (Fig. 2a,b), which could lead to unfolding/misfolding.

Next, we analysed the effect of the mutation on aggregation propensity with a high specificity prediction tool, TANGO^21^. It is important to point out that beta aggregation is mediated by short stretches that need to become exposed by (partial) unfolding before they can actually nucleate protein aggregation. The mutant residue is found flanking a very strong aggregation prone region (APR) (Fig. 2c). Normally, APRs are buried inside the hydrophobic core of the protein, however, by analysing this region in the structure it seems that this APR is solvent exposed (Fig. 2d). Exposed APRs have been correlated with protein–protein interaction surfaces^22^, hence, this APR could have a major role in the binding to microtubules. This is highlighted by the high conservation that this APR has in the MAPRE family (Supplementary Fig. S5). Other aggregation prediction tools such as AGGRESCAN^23^, PASTA 2.0^24^ and FoldAmyloid^25^ also indicated that this region is an APR (Supplementary Fig. S5). The N68S mutation slightly increases the aggregation propensity of the APR from an average score of 41.8 to an average score of 44.6. This increase itself might not be sufficient to affect the aggregation tendency of the protein, but, as the mutation is destabilizing, it could hamper the native fold by requiring higher energy states and indirectly increase aggregation (Fig. 2e), explaining the smaller abundance levels of MAPRE2 in the N68S/N68S line.

### Differentiation towards cranial neural crest lineage

Previous experiments performed by our lab using zebrafish as a model showed that the different *MAPRE2* mutations perturb the patterning of the branchial arches, indicating that the typical facial phenotype observed in the CSC-KT patients might be the result of defective neural crest migration^1^. In order to evaluate the effect of these mutations on neural crest migration using the different human pluripotent cell lines, we developed a differentiation protocol that supports a cranial neural crest identity adapted from^26,27^ (Supplementary Fig. S6). We observed an increase in gene expression of *TFAP2A*, *p75*, *SOX10* and *ETS1* (cranial neural crest marker), whereas pluripotency genes *OCT4*, *SOX2*, *Nanog*, and *PHOX2B* (trunk neural crest marker^28^) were decreased (Supplementary Fig. S6), confirming the cranial neural crest identity. This protocol favors the differentiation of cranial neural crest cells, which expresses the characteristic neural crest markers P75NTR, TFAP2A, SOX10 and SIX1. Neural crest identity was confirmed by immunostaining and RT-qPCR. Cultures for all genotypes stained positive for neural crest markers TFAP2A and p75 (Supplementary Fig. S7).

### MAPRE2 mutant cell lines show an altered migration capacity in vitro

**Figure 3:**
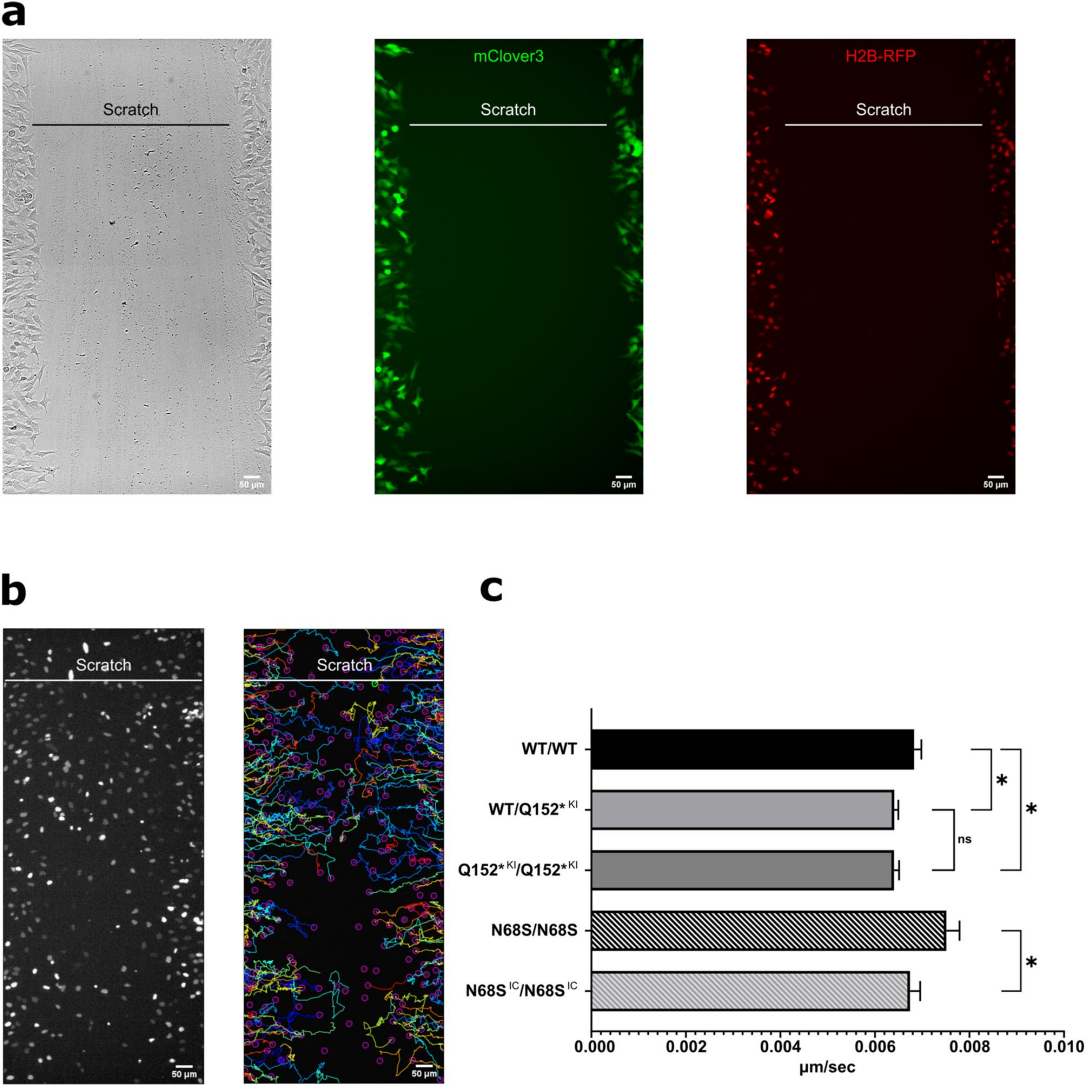
In vitro migration of iPSC derived wild type and mutant cranial neural crest cells. (**a**) Representative images of scratched area for BJ1 wild type derived CNCC to migrate into at 0 h. Cells have been transfected with mRNA 24 h prior to imaging. From left to right: Bright field, cytosolic free-floating mClover3 and nuclei labelled with H2B-mCherry. Scale bar is 50 μm. Also see Additional Movie Files 1, 2 and 3. (**b**) Left: image of the same scratched area in (**a**) after 24 h of migration visualizing labeled nuclei with H2B-mCherry. Right: Representative image of tracking the migration path of individual neural crest cells using ImageJ plugin TrackMate. (**c**) Quantification of the neural crest migration speed. Cells carrying either the heterozygous or the homozygous Q152* mutation (Q152*^KI^/WT, Q152*^KI^/Q152*^KI^) displayed a lower migration speed compared to their isogenic control line WT/WT. No difference between the full knock down Q152*^KI^/Q152*^KI^ of MAPRE2 compared to the heterozygous knock down Q152*KI/WT was noted (One-Way ANOVA, Tukey test, P = 0.045, P = 0.0423 and P = 0.9996, respectively). In contrast, the N68S/N68S mutant NC cells migrated faster than their respective isogenic control line N68S^IC^/N68S^IC^ (Unpaired t-test, P = 0.044). N = 11 for each genotype.

Given that microtubules and their associated binding proteins play an essential role in cell migration^10,29,30^, we assessed whether mutations in *MAPRE2* affect CNCC migration in vitro. To this purpose, we performed a scratch assay in combination with live imaging, enabling us to track the single cranial neural crest cells as they migrate into the open area of the scratch (Fig. 3a). One day prior to scratching, we transfected the cell cultures with H2B-RFP to label the nuclei, allowing easy tracking of single cells (Fig. 3a,b). We found that NC cells carrying either the heterozygous or the homozygous Q152* stop mutation (Q152*^KI^/WT, Q152*^KI^/Q152*^KI^), representing the loss of function status, displayed a lower migration speed compared to their isogenic control line. Interestingly, we could not detect a difference between the full knock down Q152*^KI^/Q152*^KI^ of MAPRE2 compared to the heterozygous knock down Q152*^KI^/WT, indicating that haploinsufficiency is sufficient to cause a phenotype, as seen in patients carrying these mutations (Fig. 3c, One-way ANOVA P = 0.045, P = 0.0423 and P = 0.9996, respectively). In contrast, the N68S/N68S mutant NC cells migrated faster than their respective isogenic control line N68S^IC^/N68S^IC^ (Fig. 3c, Unpaired t-test, P = 0.044). These results demonstrate that MAPRE2 is involved in cell migration in vitro. Supplemental movie files show this in more detail (Additional Movie Files 1, 2 and 3).

### MAPRE2 mutations impact the focal adhesion in cranial neural crest cells

**Figure 4:**
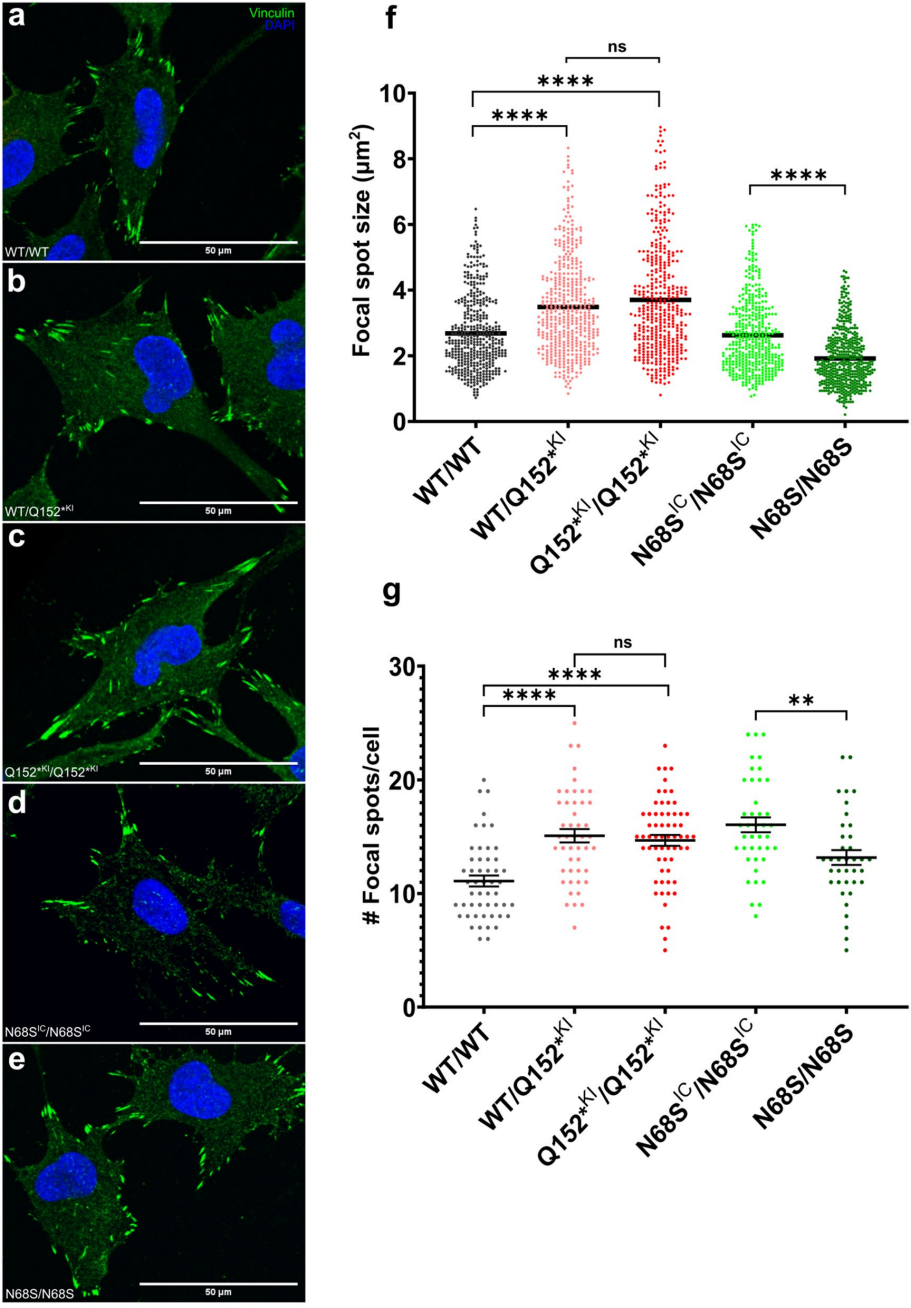
Focal adhesion in iPSC derived cranial neural crest. (**a**–**e**) Immunofluorescence staining for DAPI (blue) and focal adhesion marker vinculin (green) in iPSC derived CNCC plated into fibronectin coated culture plates. Cells were imaged using confocal microscopy. Scale bar is 50 μm. (**f**) Quantification and size distribution of FA spots. N > 400 per genotype. An increased labelling of focal adhesion spots in both Q152*^KI^/WT and Q152*^KI^/Q152*^KI^ mutant lines is observed compared to their isogenic control WT/WT with no difference between Q152*^KI^/WT and Q152*^KI^/Q152*^KI^ (Kruskal–Wallis test, P < 0.0001, P < 0.0001, P > 0.9999, respectively). Less labelling of focal adhesion spots and a decrease in spot size for the N68S/N68S mutant is observed compared to its isogenic control N68S^IC^/N68S^IC^ (Mann–Whitney test; P < 0.0001). (**g**) Quantification of number of FA spots per cell. N =  > 37 per genotype. An increase in focal adhesion spots in both Q152*^KI^/WT and Q152*^KI^/Q152*^KI^ mutant lines is observed compared to their isogenic control WT/WT with no difference between Q152*^KI^/WT and Q152*^KI^/Q152*^KI^ (One-way ANOVA, P < 0.0001, P < 0.0001, P > 0.9999, respectively). Fewer focal adhesion spots for the N68S/N68S mutant is observed compared to its isogenic control N68S^IC^/N68S^IC^ (Unpaired t test; P = 0.0026).

Microtubules can specifically grow towards focal adhesions (FA) and promote FA turnover rate at the cell periphery in order to retract and allow cell motility. It has been speculated that microtubules act as tracks to deliver factors to regulate FA turnover. Recent studies suggest that MAPRE2 acts as an adaptor to recruit a variety of signaling molecules to the microtubule ends. In this regard, it has been shown that MAPRE2 interacts with MAP4K4 and HAX1 in the FA turnover complex and influences wound healing and cell motility^9,10^. To investigate the effect of altered MAPRE2 activity on FA, we employed immunohistochemistry and confocal microscopy to visualize focal adhesion spots by the presence of vinculin in iPSC derived cranial neural crest cells plated into fibronectin-coated plates. Vinculin positive foci (focal adhesion spots) appeared larger in both MAPRE2 Q152*^KI^/WT and Q152*^KI^/Q152*^KI^ knock-down lines compared to their isogenic control WT/WT (Fig. 4a–c). Quantification of the FA spot sizes revealed a significant increase of both mutants relative to their isogenic control (Fig. 4f, Kurskal–Wallis test; P < 0.0001). Again, we observed no difference in FA spot size between the WT/Q152*^KI^ and Q152*^KI^/Q152*^KI^ mutants (Kruskal–Wallis test; P > 0.9999). As expected, smaller Vinculin positive foci and a decrease in FA spot size were seen for the N68S/N68S mutant compared to its isogenic N68S^R^/N68S^R^ control (Fig. 4d–f, Man–Whitney test; P < 0.0001). Next to FA spot size, we also looked at the number of FA spots per cell. For both MAPRE2 Q152*^KI^/WT and Q152*^KI^/Q152*^KI^ mutant lines an increase in number of FA spots compared to their isogenic control WT/WT line was noticed (Fig. 4g, One-way ANOVA, P < 0.0001, P < 0.0001, respectively). Again, we observed no difference in FA spot size between the WT/Q152*^KI^ and Q152*^KI^/Q152*^KI^ mutants (One-way ANOVA; P > 0.9999). In contrast, less FA spots were observed for the overactive N68S/N68S mutant compared to its isogenic N68S^IC^/N68S^IC^ control (Fig. 4g, Unpaired t test; P = 0.0026). Finally, as a confirmation we performed co-staining of MAPRE2 with ACF7/MACF1 encoding Microtubule Actin Crosslinking Factor 1 (MACF1) (Supplemental Fig. S8). MACF1 is a well-known crosslinking protein essential for FA dynamics and cell motility^31^. Quantification of MACF1 and Vinculin co-localization showed similar FA changes for the different mutant lines, compared to their respective controls (Supplemental Fig. S8). Together, our results provide evidence that MAPRE2 activity levels have an effect on FA turnover, and thus on CNC cell motility and migration.

### Knock down of MAPRE2 results in a defective migration capacity in vivo

**Figure 5:**
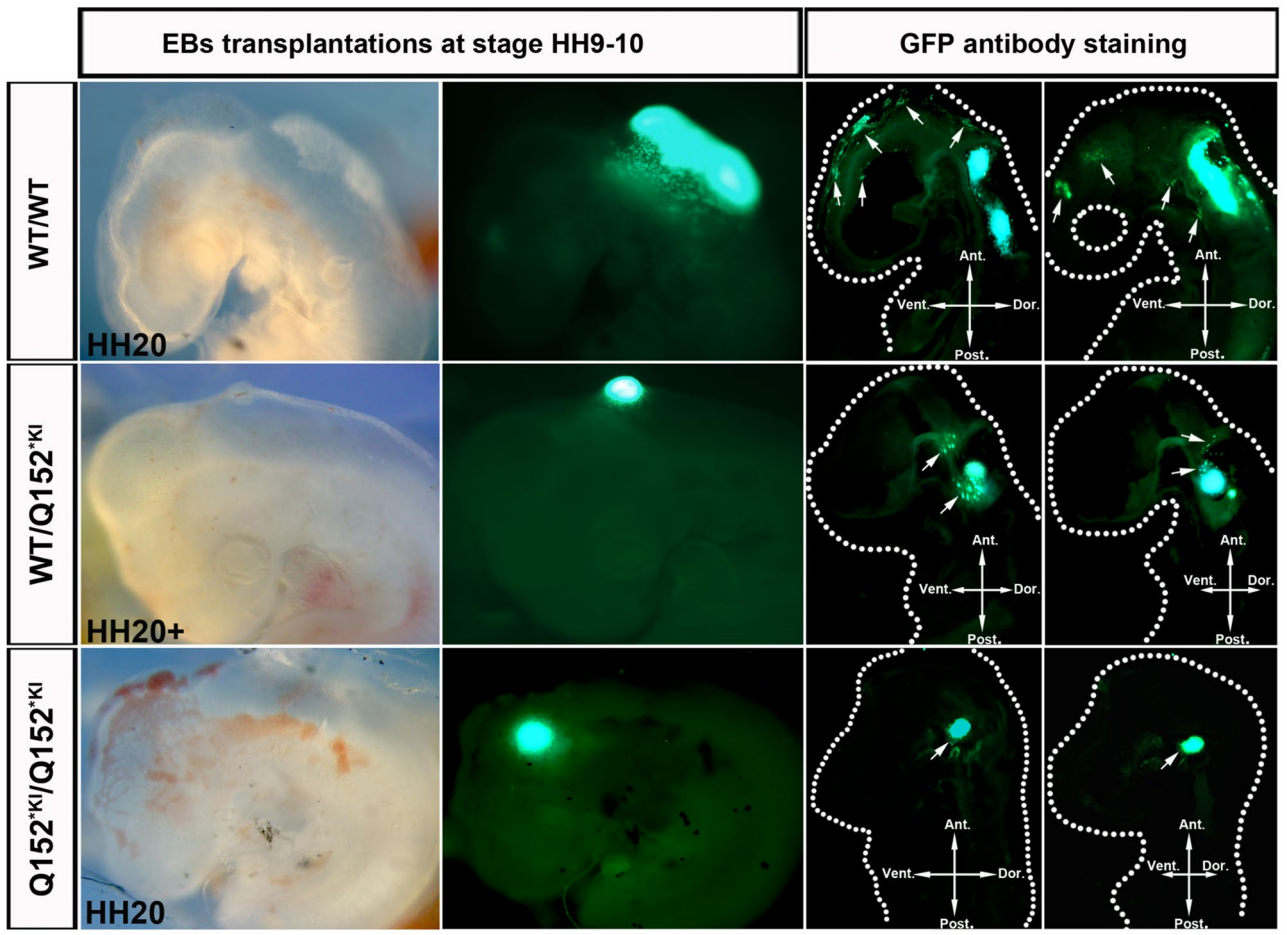
Absent migration of xenotransplanted MAPRE2 deficient neural crest progenitors in chicken embryos. Representative whole mount bright field and fluorescent images of chicken embryos at development stage HH 20 with corresponding sagittal sections. Embryos have been transplanted with EBs at day 5 of neural crest differentiation^49^, generated from EmGFP tagged MAPRE2 deficient iPSCs. Sagittal sections have been stained against endogenous EmGFP. WT/WT cells are observed migrating into the branchial arches and facial mesenchyme on whole mount and in sections, indicated by white arrows. Q152*^KI^/WT cells emerge from the EB but fail to migrate away towards the branchial arches and facial mesenchyme (indicated by white arrows). Complete MAPRE2 knock out cells (Q152*^KI^/Q152*^KI^) fail to emerge from the EB and migrate. (*Ant.* anterior, *Vent.* ventral, *Dor.* dorsal, *Post.* posterior).

Neural crest cells were first discovered in chicken embryos and later their unique features have been revealed in other avian and amphibian embryos^32–34^. Chicken embryos have been used as a model to study human stem cell potency^35,36^ and are nowadays used to study neurocristopathies^37,38^. In order to further assess the cranial migration capacity of our MAPRE2 haploinsufficient cell lines, we performed orthotopic xenotransplantation of embryoid bodies (EB) generated from eGFP labelled cells (Fig. 5)^39^. EBs were xenotransplanted into the anterior neural region between somite 8 and 10 of developing chicken embryos at Hamburger-Hamilton (HH) development stage 10. Embryos were harvested at stage HH 20, 48 h after transplantation, to assess success of the transplantation and visualize migration of human neural crest cells emigrating from the EBs. We observed that neural crest cells emerging from WT/WT control EBs clearly migrated towards the branchial arches and facial mesenchyme at stage HH19-20 (20 out of the 22 injected embryo’s, Fig. 5 top row). In contrast, neural crest cells derived from EBs from the MAPRE2 Q152*^KI^/WT line showed either migration of very low number of cells accumulating around the EB itself (16 out of 19 embryo’s) or no migration at all (3 out of 19, Fig. 5 middle row). Lastly, EBs generated from Q152*^KI^/Q152*^KI^ mutant lines did not yield any migrating cells (N = 17, Fig. 5 bottom row). Collectively, these results confirm that knock down of MAPRE2 affects migration of cranial neural crest during crucial stages of embryonic development and are likely to have a negative effect on the development of craniofacial structures.

## Discussion

Circumferential skin creases Kunze type is a rare polymalformative congenital syndrome characterized by several craniofacial defects, including a median cleft palate^1,2^. Craniofacial development begins with the migration of CNCC, a cell population unique to vertebrates, from the neural tube to the frontonasal process, first branchial arch, and supraorbital arch^40^. Normal craniofacial development culminates from a series of tightly orchestrated events, beginning with initiation and migration and ending with differentiation of specialized cell types^41^. If any of the steps of these events are even slightly altered, this can result in abnormal craniofacial morphology. Based on the role of MAPRE2 as a MT binding protein, as well as our previous studies in zebrafish that showed that *MAPRE2* mutations perturb the patterning of branchial arches, we speculated that MAPRE2 might play a role in proper migration of CNCC. In addition, it has been shown that MAPRE2 is involved in the migration and invasion of certain cancer types^42^. Both our in vitro and in vivo migration assays clearly showed altered migration capacity of *MAPRE2* mutant CNCCs. Tracking of single CNCC, with either absent or haploinsufficient amount of MAPRE2 showed a greatly diminished migration speed in vitro, while the opposite was seen for the CNCC harboring the overactive N68S missense mutation. It is known that neural crest cells have an intrinsic migratory capacity, and follow a specific pre-programmed route as soon as they detach from the neural tube. However, the local environment also plays a role as it facilitates and directs migration via several inductive signals^43^. Therefore, in order to verify whether the presence of a more physiological in vivo environment would influence the migration capacity of our MAPRE2 knock-out CNCC, we performed xenotransplantations in early developing chicken embryos. The transplanted wild type neural crest progenitors showed a normal migration of crest into the branchial arches and facial mesenchyme while transplantation of MAPRE2 partial and full knock down progenitors again showed very little to no migration. These results provide evidence that a precise amount of functional MAPRE2 protein is essential for proper CNCC migration. In addition, we observed the same conundrum as in our previous work, where the N68S mutation resulted in an overactive protein^1^, even though, as shown here, the amount of protein is significantly reduced. In our scratch assay, this mutant also behaved opposite to the knock-down cell line with a significant increase in CNCC migration speed compared to its isogenic control, yet the clinical phenotype in human is indistinguishable. Once the CNCC have arrived in the presumptive facial primordia, subsequent proliferation, differentiation and reciprocal interaction with the surrounding ectoderm and mesoderm will result in the formation of the facial prominences that make up the face^44^. However, as these processes are strictly spatio-temporally regulated, we might speculate that when the CNCC arrive too early or too late at their destination, this might both affect proper development. In addition, we cannot exclude that the mutant CNCC are unperceptive to certain environmental cues since MAPRE2 interacts with a much wider array of proteins, such as potential signaling molecules and cell cycle regulators^10^.

In order to explain this altered migration, we looked at the focal adhesions (FA) in our CNCC system. Migration depends on cell adhesion to the extracellular matrix (ECM), which occurs via FA, that connect the ECM to the cell cytoskeleton^45^. Recent studies showed that MAPRE2 is involved in FA dynamics and cell migration via close interaction with HAX1 and MAP4K4 in epidermal cells^9,10^. Depletion of either MAPRE2, HAX1 or MAP4K4 alters FA and has a negative impact on migration in epidermal cells. Similar results are seen in our CNCC where FA are enlarged and more abundant when MAPRE2 is knocked down or knocked out, while the opposite is observed in the overactive N68S variant, with significant smaller and fewer FA. It is suggested that MAPRE2 plays a role in the crosstalk between microtubules and FA as an adaptor. In the absence of functional MAPRE2 protein, fewer FAs are targeted, enhancing cell adhesion and impairing migration, as seen in our migration assays. Further studies are required to decipher the signaling pathways and the plethora of MAPRE2 interacting proteins involved in these essential developmental processes.

In conclusion, the data presented here provide further evidence that MAPRE2 is involved in cellular migration of CNCC, among other via its involvement in focal adhesion dynamics. This provides critical insights into the mechanism underlying the craniofacial malformations present in CSC-KT patients. In addition, this adds the CSC-KT disorder to the growing list of neurocristopathies.

## Materials and methods

### Reprogramming to iPSC

Fibroblasts were obtained via skin puncture and reprogrammed using non-integrating Sendai virus^46^. All lines were fully validated by analyzing the endogenous expression of pluripotent genes and by analyzing their ability to generate the three germ layers by in vitro embryoid body differentiation and teratoma formation. The presence of the respective mutation was confirmed via Sanger sequencing and comparative genomic hybridization (CGH) array excluded major genetic aberrations. (data not shown). This reprogramming was outsourced to and performed by the Stem Cell Institute Leuven (Belgium).

### Cell culture

Human iPSC lines were cultured in mTeSR1 media (STEMCELL Technologies) without antibiotics on Matrigel (Corning Life Sciences) coated culture ware as a chemically defined xeno-free cell culture matrix and maintained at 37 degrees Celsius and 5% CO_2_. Media was changed daily. Cells were grown to confluency and passaged using ReLeSR (STEMCELL Technologies) according to the manufacturer’s recommendations, and routinely checked for mycoplasma contamination (MycoAlert Mycoplasma Detection Kit, Westburg).

### Generation of CRISPR-Cas9 isogenic control and knock-in hiPSC lines

The sgRNA pair sequences were designed using the MIT CRISPR design tool (Supplementary Fig. S1) and cloned into the pX335 vector (Addgene plasmid 42335) using the NEB mutagenesis kit. Gene targeting plasmids were cloned into an in-house created vector (pRE-RMCE) that contains an EmGFP and a dual HYG(PURO)/TK selection cassette. Left and right homology arms, 1 kb long and containing the desired point mutation, were synthesized as a gBlock fragment (IDT) and inserted into the pRE-RMCE vector via Gibson assembly (HiFi, NEB). In order to avoid disrupting the genetic locus we performed scarless genome editing, by combining this double nicking approach with the PiggyBac transposase, which excises the selection cassette through recombination of the TTAA sequences contained in their integration sites (inverted terminal repeats flanked by the homology arms). We therefore designed the gene targeting plasmids to integrate next to an endogenous TTAA site. Donor template and CRISPR plasmids were introduced into the cells via electroporation using the Amaxa nucleofection system (human nucleofector kit 2 and program F-16).

After electroporation, the cells were plated in 10 cm dishes coated with Matrigel in mTeSR1 media, supplemented with 10 μM ROCK inhibitor (VWR). The cells were allowed to recover for 2–3 days before starting selection with either Hygromycin B (starting at 10 μg/mL and gradually increasing to 120 μg/mL) or Puromycin (starting at 100 ng/mL and gradually increasing to 1000 ng/mL). After 10 days, colonies were manually isolated and plated in 12-well multiwell plates, expanded and genotyped. Junction PCR analysis was used to verify correct insertion of the selection cassette and further PCR analysis was performed to determine homo- or heterozygous targeting. Additionally, digital droplet PCR (ddPCR) was performed against the EmGFP sequence in the selection cassette to exclude random integration of the donor template and Sanger sequencing was performed to verify insertion of the mutation.

Excision of the selection cassette of correctly targeted clones was performed by electroporation with a PiggyBac Transposase vector (pCMV-hyPBase, Sanger Institute). Following electroporation, cells were plated in a 10 cm dish in mTeSR1 media supplemented with 10 μM ROCK inhibitor. A negative drug selection was performed using 1-(2-deoxy-2-fluoro-B-d-arabinofuranosyl)-5-iodouracil (FIAU, 200 nM) to select for cells in which the selection cassette was successfully removed. Surviving clones after 10 days were isolated and genotyped with the same methods as described above. Lastly, correction or insertion of the mutation was confirmed by Sanger sequencing (Supplementary Fig. S2) and pluripotency was assessed by staining for pluripotency markers (OCT4 and SOX2) (Supplementary Fig. S3). Comparative genome hybridisation was performed after every major step and no major genetic aberrations were found, confirming that genomic integrity was preserved after CRISPR/Cas9 treatment.

### Model generation and effect of mutant on protein stability

A homology model of MAPRE2 was built using the Swiss-model webserver (template PDB: 1vka.pdb)^19^. The model was refined using the RepairPDB function in FoldX^20^. The function BuildModel in FoldX was used to predict the impact of the N68S mutation on the thermodynamic stability of the protein. YASARA was used for visualization and to analyse residue interactions^47^.

### Prediction of aggregation prone regions

Potentially aggregating regions of MAPRE2 were predicted computationally using a combination of online prediction tools: TANGO (http://tango.crg.es/)^21^, AGGRESCAN (http://bioinf.uab.es/aggrescan/)^23^ PASTA 2.0 (http://old.protein.bio.unipd.it/pasta2/)^24^ and FoldAmyloid (http://bioinfo.protres.ru/fold-amyloid/)^25^. All parameters in each stated software were set to default and aggregating regions were determined accordingly based on their score.

### Differentiation of iPSC’s towards cells with neural crest identity

Human iPSCs were detached and single cell suspended using Stempro Accutase (Thermofisher). Cells were counted and plated at a density of 10^5^ cells/cm^2^ in Matrigel coated wells in mTeSR1 medium. The next day the medium was changed to neural crest medium (Essentials 6 medium supplemented with 20 μM SB431542 (STEMCELL technologies)), 2 μM CHIR99021 (STEMCELL technologies), 8 ng/mL bFGF and 10 ng/mL Recombinant Human Heregulinβ-1 (Tebu BIO)). Cultures were split with Stempro Accutase every 4 days for 3 weeks and reseeded at a density of 10^5^ cells/cm^2^ in Matrigel coated wells. After 4 days in culture, a double morphology (mixture of elongated and colony-like cells) is obvious and after 8 days of differentiation, early neural crest migrates away from the edges of reformed colonies. After 21 days of differentiation, the early neural crest cells were purified for neural crest surface marker p75 using the magnetic bead separation system (MACS) from Miltenyi Biotec. Shortly, the neural crest cells were detached using Stempro Accutase, pelleted and incubated with p75 magnetic beads for 15 min at 4 °C. After incubation the cells were washed, ran through a magnetic separation column using gravity flow, reseeded in neural crest medium in Matrigel coated wells and incubated at 37 °C and 5% CO_2_. The cultures were then matured until day 28. At this point, the cells were either cryopreserved or maintained by passaging every 4 days. Neural crest identity was verified at timely intervals^26,27^.

### Quantitative real-time polymerase chain reaction (qRT-PCR)

Total RNA was extracted by using Maxwell^®^ RSC simplyRNA Cells Kit (Promega). cDNA was synthesized using the SuperScript^®^ III First-Strand Synthesis System (ThermoFisher) from 3000 ng total RNA, using oligo(DT)_20_ as a primer. qRT-PCR reactions mixtures were prepared with SYBR Green Master Mix (Roche) with an input of 20 ng cDNA per reaction and analyzed on a lightcycler LC480 (Roche). Gene expression values were normalized to housekeeping gene *GAPDH* (glyceraldehyde 3-phosphate dehydrogenase). A list of primers that were used is provided in Supplementary Table S1.

### Immunocytochemistry

Cells were fixed using 4% Paraformaldehyde (PFA) for 15 min at room temperature, permeabilized with 0.3% Triton X-100 in PBS for 10 min at room temperature and blocked with 0.1% Triton X-100 in PBS supplemented with 3% BSA for 30 min at room temperature. Samples were incubated with primary antibodies overnight at 4 °C and with secondary antibodies (Alexa Fluor) the next day for 1–2 h at room temperature, protected from light. Nuclei were counter-stained using DAPI (Thermofisher). Stained coverslips were mounted onto glass slides using Coulter mounting media and sealed with acryl nail polish. Slides were immediately imaged using confocal microscopy (Nikon NiE upright A1R + HD resonant scanning upgrade) or stored at 4 °C and protected from light. A list of the antibodies is provided in Supplementary Table S1.

### In vitro migration assay

At day 0, cranial neural crest cells were dissociated with Stempro Accutase and plated in Matrigel coated 24-well plates at a density of 200,000 cells per well in order to form a monolayer. The day after, cranial neural crest cells were transfected with mClover3 and H2B-mCherry2 mRNA using Lipofectamine™ Stem Transfection Reagent (Life Technologies). Briefly, reaction mixes were made with each reaction containing 250 ng of mRNA per well and incubated at 37 °C and 5% CO_2_. After 3 h, neural crest media was added and cultures were incubated overnight. On day 2, the monolayer was scratched using a P1000 pipet tip, cell debris was removed by washing with neural crest medium and fresh media was added. Three hours after introducing the scratch, the plates were imaged every 10 min over a time course of 24 h using an Operetta microscope system. Temperature was kept at 37 °C and 5% CO_2_. Single cell migration tracking analysis was performed in ImageJ (V2.0.0) using the plugin TrackMate (V5.2.0). Images were preprocessed to only visualize the scratched area in order to accurately track the migration of single cells. Tracking on all genotypes was automated using a fixed set of parameters in TrackMate to calculate the average migration speed.

### Tagging of iPSC’s with eGFP and xenotransplantation into chicken embryos for in vivo cell migration

Stable GFP expression was achieved by introducing a GFP cassette into the AAVS1 locus using TALENs. Human iPSCs were electroporated using the Amaxa nucleofector system as described above with left, right and donor template AAVS1 TALEN plasmids^48^ (Addgene 59025, 59026 and 80945, respectively). Shortly, cells were electroporated with 2.5 μg of AAVS1 TALEN L, 2.5 μg of AAVS1 TALEN R and 5 μg of AAVS1-Pur-CAG-EGFP plasmids. After electroporation, the cells were plated in 10 cm dishes coated with Matrigel in mTeSR1 media, supplemented with 10 μM ROCK inhibitor. The cells were allowed to recover for 2–3 days before starting selection with puromycin (starting at 100 ng/mL and gradually increasing to 1000 ng/mL). After 10 days, single colonies were manually isolated into 12-well multi well plates, expanded and genotyped using the methods described above. Digital droplet PCR confirmed insertion in both alleles and fluorescent microscopy confirmed stable GFP expression over a longer period of time.

Embryoid bodies (EB) were made as described^49^. Briefly, iPSC colonies were detached using Collagenase type IV (ThermoFisher) and re-plated into uncoated tissue culture plates in neural crest induction medium to form EBs. Media and culture plates were changed daily. EBs obtained at day 5 of differentiation were injected into the developing chicken embryo in the anterior neural region between somite 8 and 10 at stage HH10. A minimum of 15 injections per genotype was performed and scored. Following microsurgery, operated eggs were sealed with medical tape and re-incubated at 37 °C and 80% humidity in a normal poultry egg incubator until stage HH20 for analysis under a fluorescence stereo microscope (Olympus SZX16). Fertilized chicken eggs (Gallus gallus domesticus) were obtained from a local breeder (LSL Rhein-Main).

### Assessment of focal adhesion

Cranial neural crest cells were plated on glass coverslip coated with fibronectin (10 μg/mL) at a density of 50,000 cells per well in a 12-well multi well plate. The cells were fixed using 4% PFA and stained for Vinculin (Sigma) and MACF1 (ThermoFisher) using the staining procedure described above. The size and number of focal adhesion spots were measured using ImageJ. Colocalisation and Pearson correlations were measured using the Coloc2 plugin in ImageJ.

### Statistical analyses

Statistical analysis was performed in Graphpad Prism (version 8). One-way ANOVA (Bonferroni multiple comparisons test) and two-tailed unpaired student’s t-test were used to determine statistical significance between groups. The results are presented as mean ± SEM and P < 0.05 values were considered as statistically significant. *P < 0.05; **P < 0.01; ***P < 0.001.

### Ethics declarations

All methods were carried out in accordance with guidelines and regulations of KU Leuven, and all experimental protocols were approved by the KUL/UZ ethical committee. More specifically, the KUL/UZ ethical committee approved the derivation of induced pluripotent stem cells from skin biopsy (performed by HVE), as well as all experiments that utilized human stem cells during this project (S57879). The parents of patient M2 consented for the skin biopsy and signed the S57879 related informed consent. According to German animal care guidelines, no IACUC (Institutional Animal Care and Use Committee) approval was necessary to perform chicken embryo experiments at this early embryonic stage.

## Supplementary Information

Supplementary Information 1.

Supplementary Video 1.

Supplementary Video 2.

Supplementary Video 3.

## Data Availability

The datasets used and/or analyzed during the current study are available from the corresponding author on reasonable request.
