# Supplementary material for: MAPRE2 mutations result in altered human cranial neural crest migration, underlying craniofacial malformations in CSC-KT syndrome
Source: Sci Rep. 2021 Mar 2;11:4976. doi: 10.1038/s41598-021-83771-3 (PMC7925611; doi:10.1038/s41598-021-83771-3)
Supplement: Supplementary file 1 — Supplementary Information 1. [file 41598_2021_83771_MOESM1_ESM.pdf]

SUPPLEMENTAL INFORMATION

Title

MAPRE2 mutations result in altered cranial neural crest migration, underlying circumferential skin creases syndrome, Kunze type

Author list

Cedric Thues, Jorge S. Valadas, Liesbeth Deaulmerie, Ann Geens , Amit K. Chouhan , Ramon Duran-Romaña, Joost Schymkowitz, Frederic Rousseau, Michaela Bartusel, Rizwan Rehimi, Alvaro Rada-Iglesias, Patrik Verstreken, Hilde Van Esch

Additional movie file 1 Bright filed movie of wild type derived neural crest cells migrating into the scratched area during *in vitro* migration assay.

Additional movie file 2 Movie of Mclover3 channel of wild type derived neural crest cells migrating into the scratched area during *in vitro* migration assay.

Additional movie file 3 Movie of mCherry3 H2B channel of wild type derived neural crest cells migrating into the scratched area during *in vitro* migration assay.

Additional table 1. List of primers and antibodies

Primer name	Sequence
qPCR MAPRE2 exon 6 Fw	CTCTTCCCTGGCTGCATTAG
qPCR MAPRE2 exon 6 Rv	CGCTTAAATGATGCTTGCAG
qPCR MAPRE2 exon 8 Fw	TCCAGCAGCTAAACCAGGAT
qPCR MAPRE2 exon 8 Rv	AGCTGTATGACCTGCGTTTCT
GAPDH Fw	TCAAGAAGGTGGTGAAGCAGG
GAPDH Rv	ACCAGGAAATGAGCTTGACAAA
MAPRE2 sequencing exon 1 Fw	GGCAGGAAGAGGTCACTAAAAAT
MAPRE2 sequencing exon 1 Rv	TAAGGAAGCATTTCATTTCCAGA
MAPRE2 sequencing exon 2 Fw	AAAACTGGTTCTGCTGCTAACTG
MAPRE2 sequencing exon 2 Rv	GAAAAGAAAACCTGCCTTTTGAT
MAPRE2 sequencing exon 3 Fw	GACTTTTGGATATTTGGCGTGTA
MAPRE2 sequencing exon 3 Rv	TAATTAGCCCTTCGTACACTTGG
MAPRE2 sequencing exon 4 Fw	GAGAGCTGGGAGAAGGCAGT
MAPRE2 sequencing exon 4 Rv	ACCTATTCACAAAATGCTTTCCA
MAPRE2 sequencing exon 5 Fw	CAGAACCTGAGGAACATTTGG
MAPRE2 sequencing exon 5 Rv	TCTCAGCAAAGGAACCATGA
MAPRE2 sequencing exon 6 Fw	GTACAGTCCCTTCCCTCTAGCTC
MAPRE2 sequencing exon 6 Rv	CAGCCTACTGTTGGGATATCAAG
MAPRE2 sequencing exon 7 Fw	AAACAGTGCTGCCACAAACTT
MAPRE2 sequencing exon 7 Rv	CTGGTCTGTACTGCCAGCAA
MAPRE2 sequencing exon 8 Fw	TGAGATAAGTTGCCCAAAACAAC
MAPRE2 sequencing exon 8 Rv	CATGGGCTACTGATTTTGTCTTC
MAPRE2 sequencing exon 9 Fw	CCTGTTGTTTCTCCCTCAGACTT
MAPRE2 sequencing exon 9 Rv	ACGATGTTTATTCCTTTGGGACT
MAPRE2 sequencing exon 10 Fw	TGATCTGTTCTTTGATCACATGG
MAPRE2 sequencing exon 10 Rv	GTATGTGACCCTACGATCTCTCG
M2 left junction assay Fw	CCGTCCCATTAGAACTTGAATAA
M2 left junction assay Rv	TACATTCTAGGTCGGGGTTTGTA
M2 right junction assay Fw	GCAATAGCATCACAAATTTCACA
M2 right junction assay Rv	CCAAAGGAGGACTGCTTATTTCT
M2 cell PCR Fw	GGACTTTTACATGCCCTGGA
M2 cell PCR Rv	AACAAAACGCAGTCCTGTCC
M1 left junction assay Fw	ATCTCAACACAACTGGGCTACAT
M1 left junction assay Rv	AAATCAGTGACACTTACCGCATT
M1 right junction assay Fw	AACCCCGACGGTACTAAAAGTT
M1 right junction assay Rv	GGTTCTCTTTCAGCACTTCTTGA
M1 cell PCR Fw	AAACAGTGCTGCCACAAACTT
M1 cell PCR Rv	CTGGTCTGTACTGCCAGCAA
qPCR OCT4 Fw	TCGAGAACCGAGTGAGAGG
qPCR OCT4 Rv	GAACCACACTCGGACCACA
qPCR SOX2 Fw	GAGTGGAAACTTTTGTCGGAGA
qPCR SOX2 Rv	AGCGTGTACTTATCCTTCTTCAT
qPCR p75 Fw	CCCTGTCTATTGCTCCATCC
qPCR p75 Rv	GCTCCTTGCTTGTTCTGCTT
qPCR TFAP2A Fw	ATGCTTTGGAAATTGACGGA
qPCR TFAP2A Rv	ATTGACCTACAGTGCCCAGC

qPCR PHOX2B Fw	GGAGACTCACTACCCCGACA
qPCR PHOX2B Rv	CTCCTGCTTGCGAAACTTGG
qPCR ETS1 Fw	CCAGACTTTGTTGGGGACAT
qPCR ETS1 Rv	TCTGGATAGGCTGGGTTGAC
qPCR SOX10 Fw	TACCCGCACCTGCACAAC
qPCR SOX10 Rv	TTCAGCAGCCTCCAGAGC

**Name**	**Supplier**	**Cat#**	**RRID**
OCT4	Abcam	ab19857	AB_445175
SOX2	Bio-connect	sc-365823	AB_10842165
NGFR p75	Sigma Aldrich	N3908	AB_260763
TFAP2A	Sigma Aldrich	HPA028850-100UL	AB_10600730
GAPDH	Abcam	ab8245	AB_2107448
B tubulin	Imtec	802001	AB_2564645
MAPRE2	Sigma Aldrich	HPA016739	AB_1853570
Vinculin	Sigma Aldrich	V9131-100UL	AB_477629
MACF1	Thermo Fisher	PA5-66976	AB_2665172

Supplemental Fig. S1

a

	OCT4		SOX2		Nanog	
	Ct Mean	ΔCt GAPDH	Ct Mean	ΔCt GAPDH	Ct Mean	ΔCt GAPDH
N68S/N68S	15,741	1,589	18,698	4,544	19,665	5,512

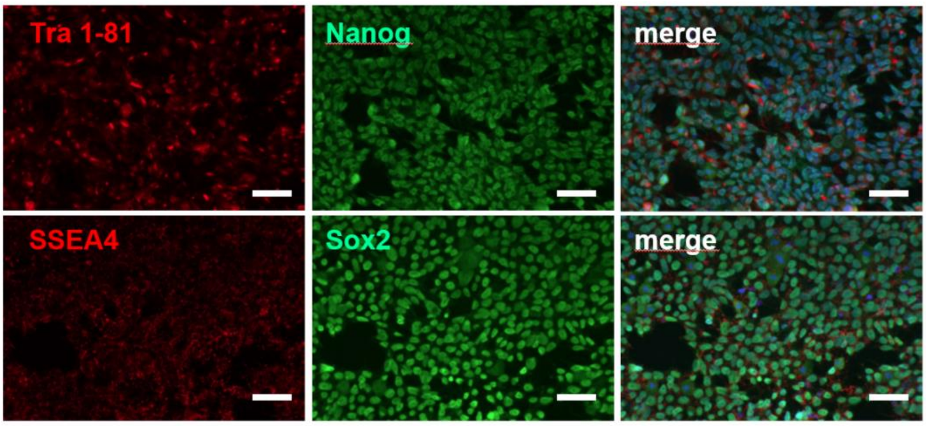

b

	Self-renewal	Ectoderm	Mesoderm	Endoderm
N68S/N68S stem cells	- 0,83	0,04	-0,15	-1,38
N68S/N68S EB	- 6,32	2,01	1,79	2,46

x>1.5	Upregulated
1.0<x<=1.5	
0.5<x<=1.0	
-0.5<=x<=0.5	Comparable
-1.0<=x<-0.5	
-1.5<=x<-1.0	
x<-1.5	Downregulated

Gene expression relative to the reference standard

c

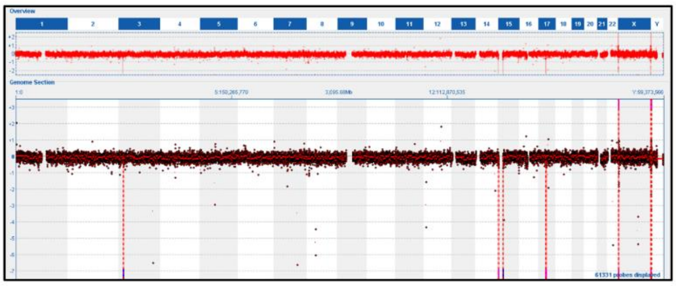

N68S/N68S fibroblasts

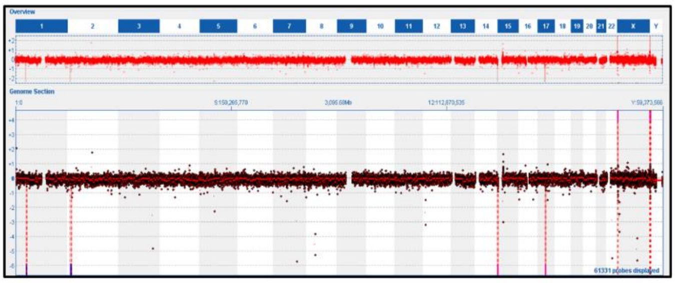

N68S/N68S iPSCs

Supplemental Fig. S1 Characterization of N68S/N68S patient derived iPSCs. **a** N68S/N68S derived iPSC clones express typical pluripotency markers as SOX2, Nanog, OCT4, TRA 1-81 and SEEA4, as shown by qPCR and immunostaining. Hoechst 33258 (blue) was used as nuclear marker. Scale bars: 50 µm. **b** Results of embryoid body (EB) formation and characterization using the score card assay (three germ layer differentiation and loss of pluripotency marker gene expression). **c** Whole genome array comparative genome hybridisation performed on DNA from the original patient fibroblasts and the derived N68S/N68S iPSC line, showing stable reprogramming.

Supplemental Fig. S2

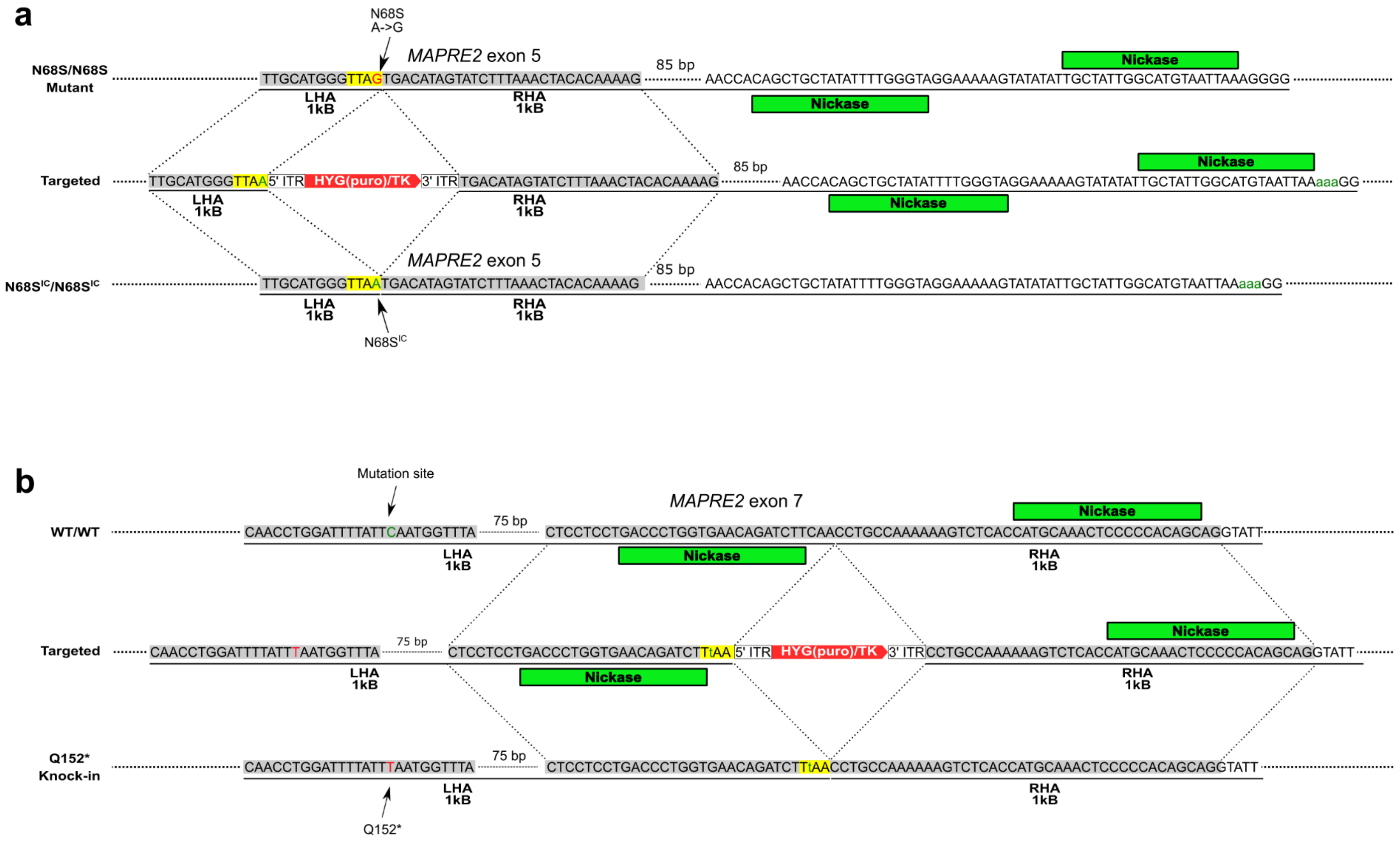

Supplemental Fig. S2 Detailed CRISPR/Cas9 strategy for correction of N68S and knock-in of Q152* mutations. **a** Detailed schematic of CRISPR/Cas9 strategy to correct the homozygous N68S/N68S mutation in patient derived iPSCs. Note the exonic regions are highlighted in grey and the CRISPR cut site is 148 bp away from the mutation site. Additionally, correction of the N68S mutation generates a TTAA site, which will be used afterwards to excise the selection cassette with PiggyBac Transposase. Lastly, one of the two PAM sequences is mutated to prevent re-cutting of the Cas9 proteins. **b** Detailed schematic of CRISPR/Cas9 strategy to introduce the premature stop codon Q152*^{KI} into a wild type background. Note the exonic region is highlighted in grey and the CRISPR cut site is 125 bp away from the mutation site. A silent mutation is introduced to create an endogenous TTAA site for excision and the selection cassette is introduced between the two gRNA sites to prevent re-cutting of the genome.

Supplemental Fig. S3

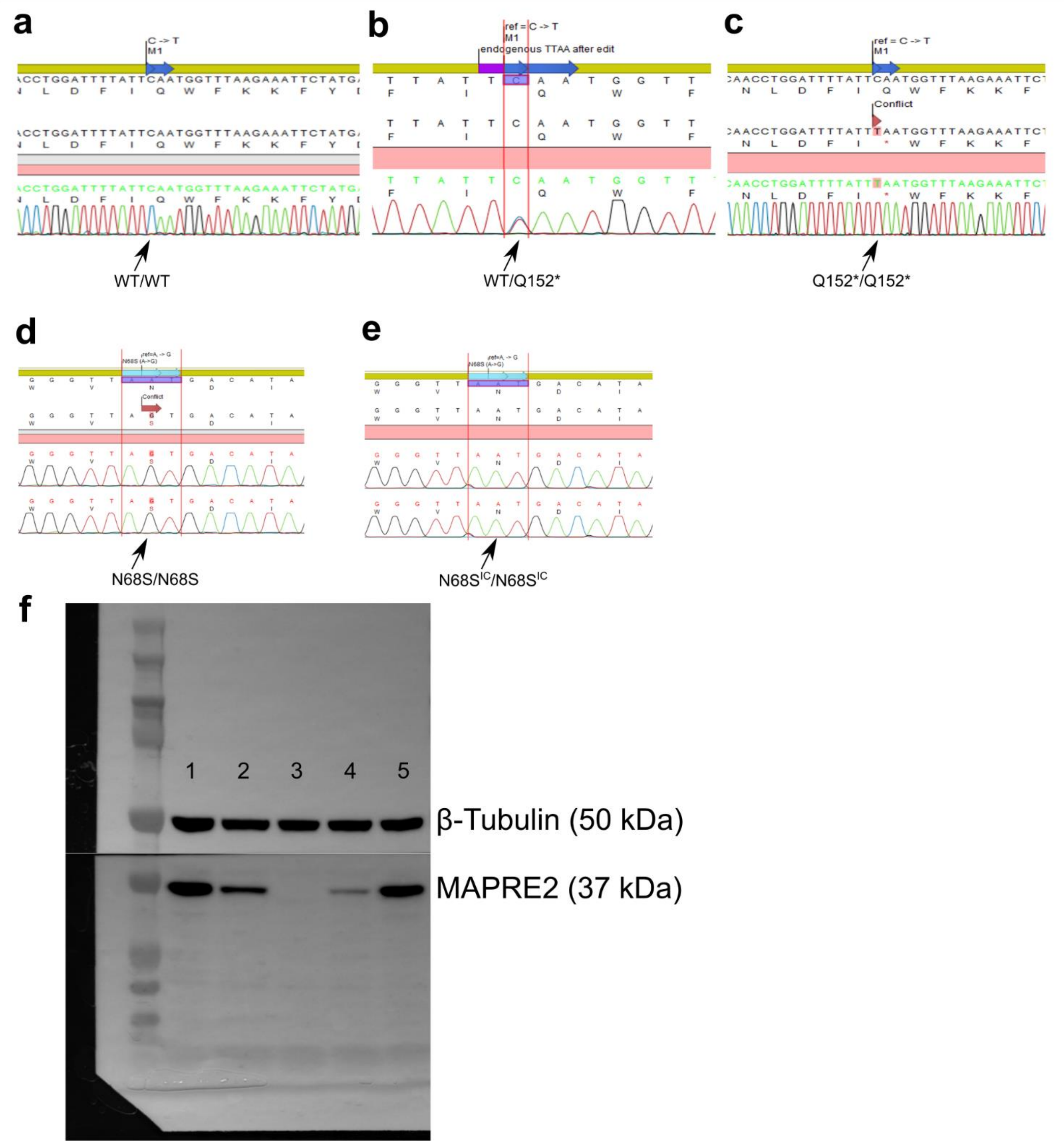

Supplemental Fig. S3 Sanger sequencing confirmation of *MAPRE2* mutant cell lines and Western blot of MAPRE2 protein expression in human iPSC. **a** Sanger sequencing of the WT/WT cell line in which the Q152* mutation will be introduced. **b** Sanger sequencing of the Q152*^{KI}/WT Bj1 background cell line. Note the heterozygosity of the mutation by the overlap of two reads. **c** Sanger sequencing of the Q152*^{KI}/ Q152*^{KI} Bj1 background cell line. **d** Sanger sequencing of N68S mutation in patient M2 (N68S/N68S) derived iPSCs. **e** Sanger sequencing of N68S^{IC}/N68S^{IC} isogenic control iPSCs. **f** Western blot image of MAPRE2 protein expression in human iPSC. Ladder was taken in bright field. β-Tubulin ECL detection was exposed for 20 seconds. MAPRE2 ECL detection was exposed for 30 seconds due to low concentration of target protein. (1. WT/WT 2. WT/Q152*^{KI} 3. Q152*^{KI}/Q152*^{KI} 4. N68S/N68S 5. N68S^{IC}/N68S^{IC}).

Supplemental Fig. S4

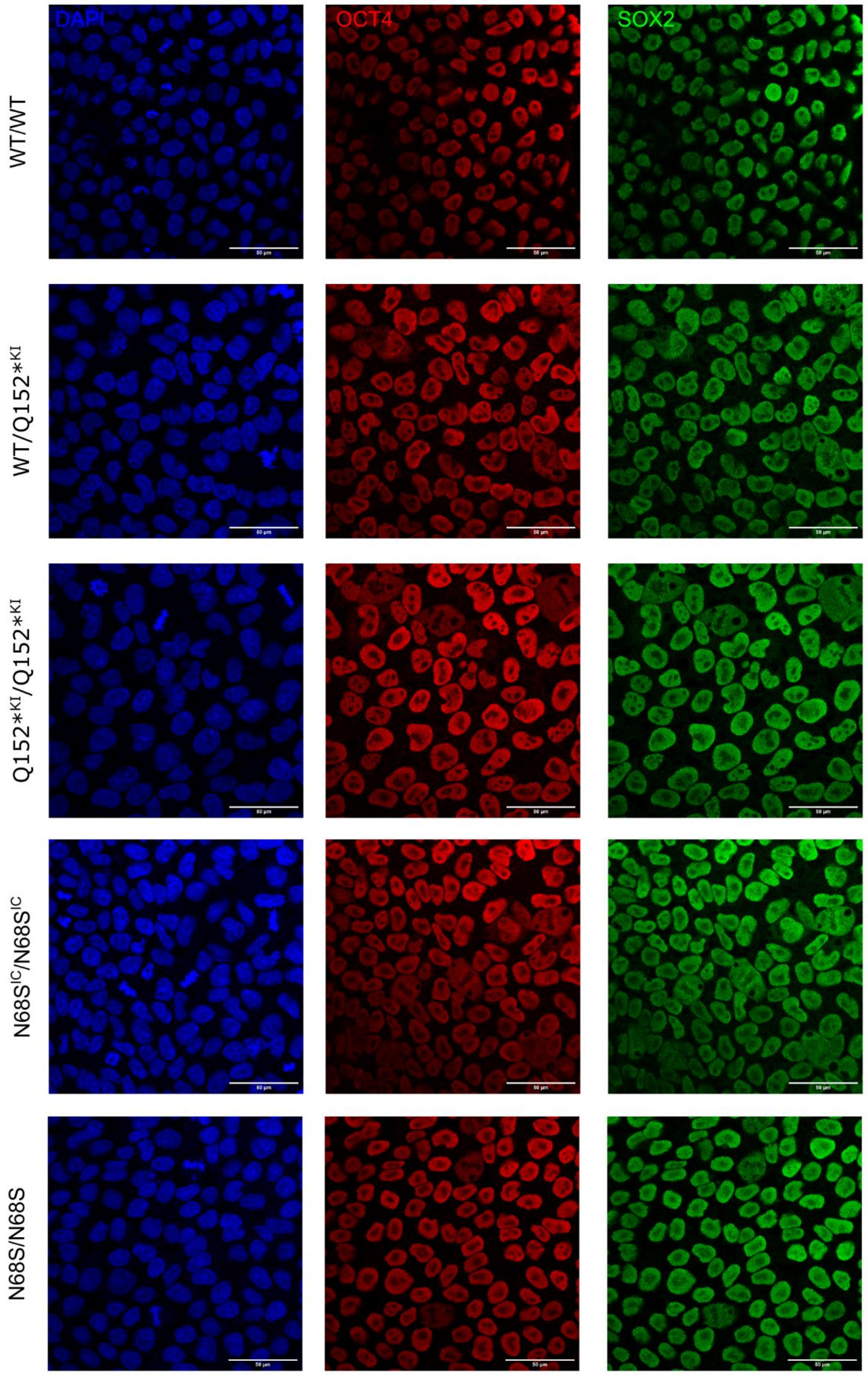

Supplemental Fig. S4 Pluripotency validation staining of MAPRE2 induced pluripotent stem cell lines. Representative immunofluorescence images of human iPSC. Immunostaining against DAPI (blue) and pluripotency markers SOX2 (green) and Oct4 (red). Scale bar is 50 µm.

Supplemental Fig. S5

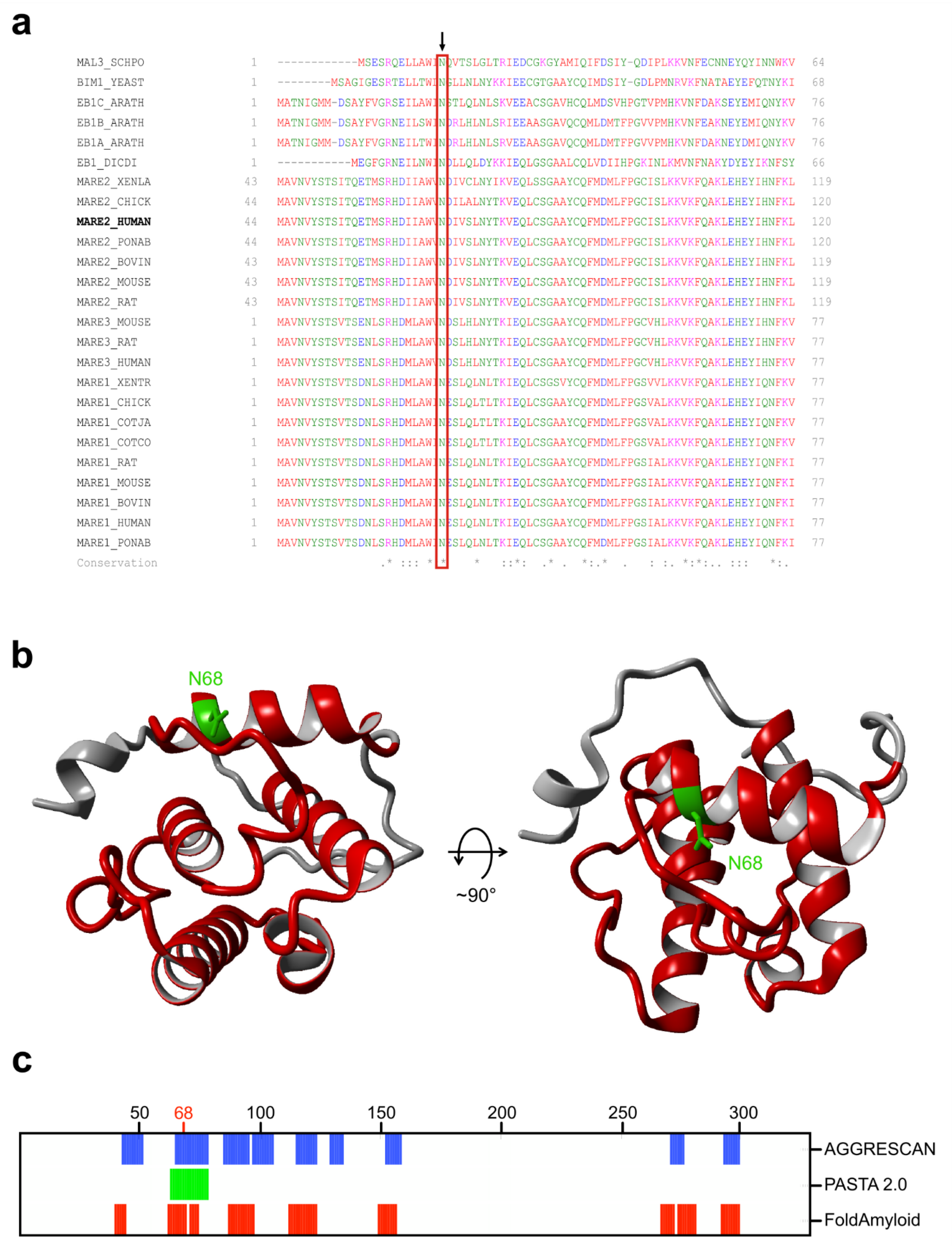

Supplemental Fig. S5 Protein structure and conservation of MAPRE2. **a** Multiple sequence alignment of all 25 proteins classified as MAPRE family in UniProt. Only a small part of the sequence for each protein is shown. The red box the position of the highly conserved mutant residue. **b** Overview of the model structure of MAPRE2 with Calponin homology domain in red, Asn 68 residue in green and the rest in grey. **c** Aggregation propensity prediction using AGGRESCAN, PASTA 2.0 and FoldAmyloid.

Supplemental Fig. S6

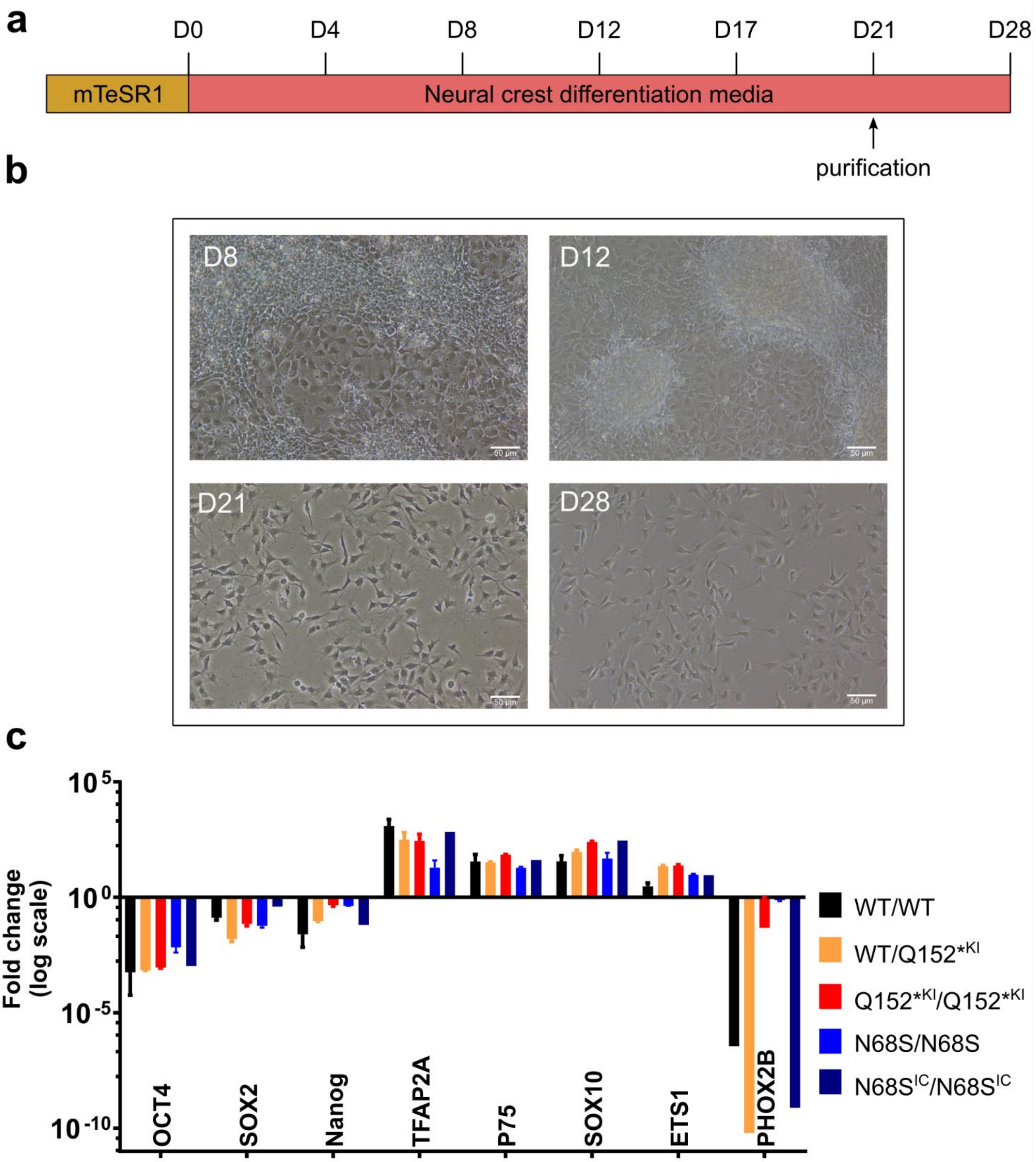

Supplemental Fig. S6 Cranial neural crest differentiation protocol. **a** Timeline of our novel cranial neural crest differentiation protocol. **b** Bright field images during cranial neural crest differentiation of the WT/WT cell line. Scale bar is 50 µm. **c** Gene expression fold changes in cranial neural crest compared to the iPSC stage after 28 days of differentiation (n=3). Note decrease in expression of pluripotency genes and upregulation of neural crest specific genes *TFAP2A*, *P75*, *SOX10*. Additionally, the expression of *ETS1* is upregulated while *PHOX2B* is downregulated, indicating the cranial identity.

Supplemental Fig. S7

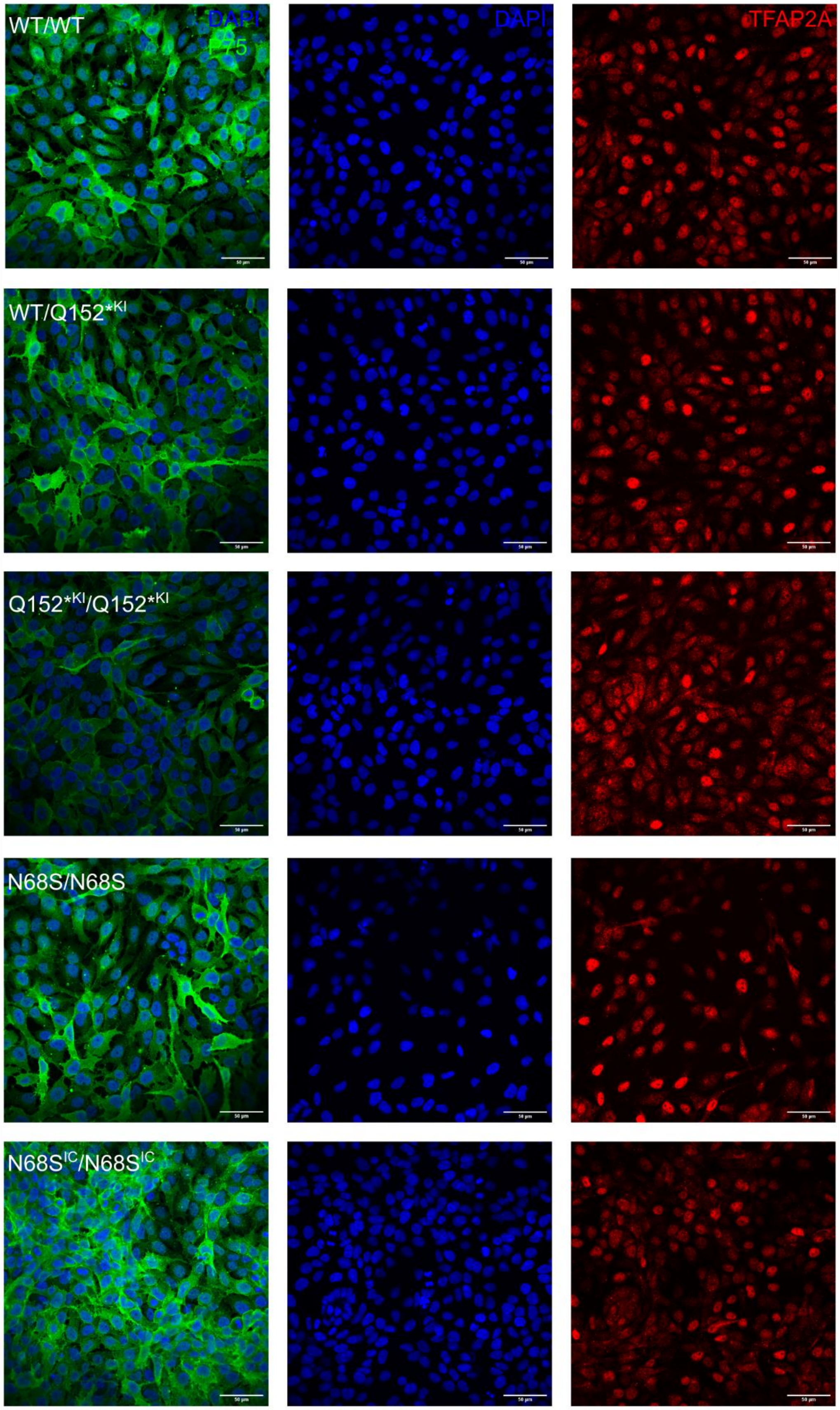

Supplemental Fig. S7 Cranial neural crest staining. Representative immunofluorescence images of human iPSC derived cranial neural crest cells. Immunostaining against DAPI (blue), P75 (green) and TFAP2A (red). Scale bar is 50 µm.

Supplemental Fig. S8

a

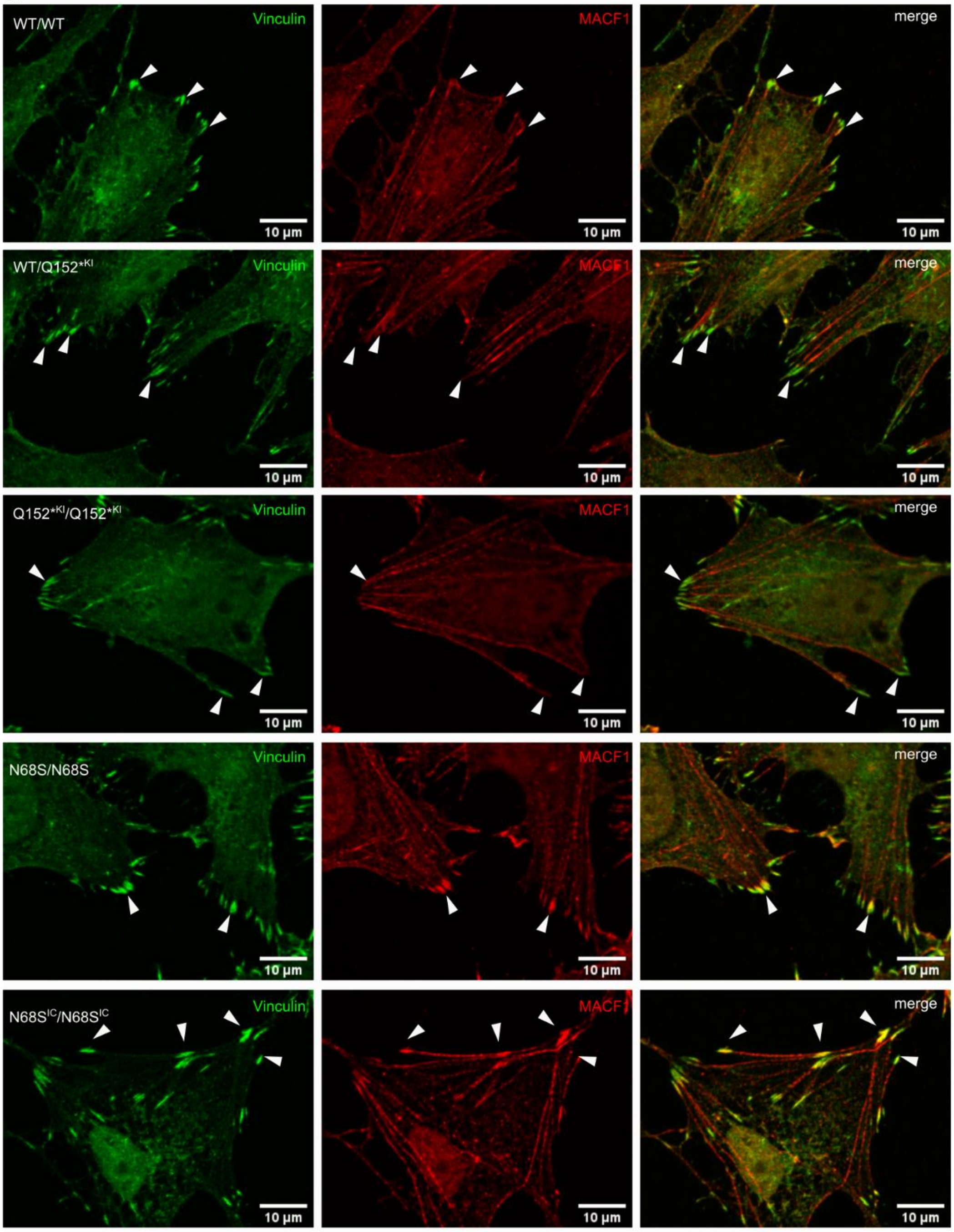

b

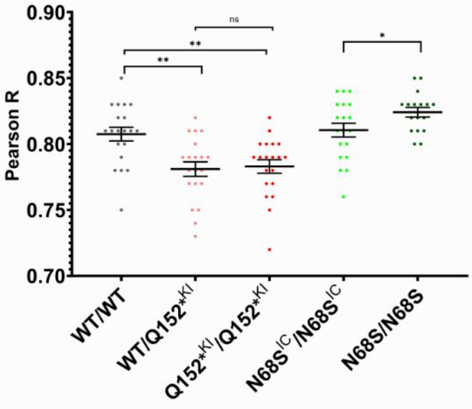

Supplemental Fig. S8 Colocalisation of focal adhesion markers in cranial neural crest. **a** Repesentative confocal immunofluorescent images of human iPSC derived cranial neural crest cells. Immunostaining against Vinculin (green) and Macf1/Acf7 (red). White arrows indicate focal adhesion spots. Scale bar is 10 µm. **b** Quantification of Vinculin and MACF1 colocalisation determined by Pearson correlation coefficient (n = >17 cells per genotype). Note the reduction in colocalisation in the abscence of MAPRE2 and the increase in colocalisation for N68S/N68S MAPRE2 mutant.
